# The Effect of Various Types of Polymeric Packaging Materials on the Quality of Copioba Cassava Flour

**DOI:** 10.3390/ma18204768

**Published:** 2025-10-17

**Authors:** Andrea Limoeiro Carvalho, Fabiane Cerqueira de Almeida, Lucas Guimarães Cardoso, Ederlan de Souza Ferreira, Geany Peruch Camilloto, Carolina Oliveira de Souza

**Affiliations:** 1 Department of Chemical Engineering, Polytechnic School, Federal University of Bahia, Prof. Aristides Novis Street, Salvador 40210-630, BA, Brazil; 2 Graduate Program in Food Science, College of Pharmacy, Federal University of Bahia, Salvador 40170-115, BA, Brazil; fabi_fca@hotmail.com (F.C.d.A.); ederlan.ferreira@ufba.br (E.d.S.F.); 3 School of Exact and Technological Sciences, Salvador University, Salvador 41720-200, BA, Brazil; 4 Department of Bromatological Analysis, College of Pharmacy, Federal University of Bahia, 147 Barão de Jeremoabo Street, Salvador 40170-115, BA, Brazil; 5 Department of Technology, State University of Feira de Santana, Transnordestina Av, Feira de Santana 44036-900, BA, Brazil

**Keywords:** *Manihot esculenta*, product identity, packaging materials, storage

## Abstract

This study assessed the impact of commercial packaging on the stability and identity of Copioba cassava flour. Flour was packaged in low-density polyethylene (LDPE), polypropylene (PP), and metallized biaxially oriented polypropylene (BOPP) films. Quality changes over time were evaluated via moisture content, water activity (*a*_w_), pH, titratable acidity, texture/hardness, color, fatty acid composition, and specific microorganisms. Moisture content and *a*_w_ increased in the LDPE-packaged flour and the control group. At the end of the storage period, levels of fatty acids had decreased by 55.81–68.28%, with only minor changes in *a*_w_. There was a rise in yeast and mold levels up to 4 log CFU/g in flour packaged in LDPE films. In contrast, the levels of *Bacillus cereus* in flour packaged in PP and BOPP ranged from <1 to 2.30 log CFU/g. PP and BOPP films exhibited the most effective performance among the packaging materials evaluated. The results obtained in this study will contribute to the pursuit of a Geographical Indication GI certification by providing information about the best packaging type for preserving the unique characteristics of Copioba cassava flour, as no study has previously reported on the best type of packaging material for Copioba flour.

## 1. Introduction

Cassava (*Manihot esculenta* Crantz) is a root crop widely grown in tropical and subtropical regions, particularly in low-income populations that face food deficits. The global production of cassava was over 330 million tons in 2023, with Nigeria being the largest producer with 62.69 million tons, and Brazil the fifth largest producer with 18.51 million tons [1]. Bahia is the biggest producer in the northeastern region of Brazil and the eighth in the country, with a production of over 800 thousand tons, most of which are in the Copioba Valley [2].

The Copioba cassava flour has distinctive sensory attributes, including fine and crispy granulation, and a white-yellowish color. These attributes emanate mainly from its processing method, which involves continuous mixing during roasting, varieties used, which are assorted because of the tradition passed by the native indigenes ancestors of the region of cultivating the cassava for different purposes, according to their characteristics [3,4,5]. This specific type of cassava flour is regarded as of high quality, associated with the traditional way of production of the region where it is produced, the Copioba Valley, Bahia, northeast of Brazil. It is classified as a product with a particular identity, one of the conditions to obtain a Geographical Indication (GI) [4,6,7].

Securing a GI requires a thorough understanding of the traditional methods involved in flour production and the safety protocols related to the production and storage processes. These include social, cultural, environmental, and historical elements that protect producers and consumers while certifying the unique qualities that define the product [8,9]. The INPI [10] has already recognized three regions in Brazil that produce cassava flours with specific characteristics associated with traditional practices inherited from the native indigenous habitats of those regions and has given them a GI certification of Indication of Origin.

A major problem with the commercialization of this product is related to its lack of safety guarantee, owing to loss of its sensory attributes and microbiological safety assurance, and inappropriate packaging for storage [4]. This challenge, which has compromised the sustainability of its production chain, especially as the market for this product expands and increasing instances of improper packaging, needs urgent attention.

The demand for Copioba cassava flour has increased as consumers turn to traditional products. Thus, there is a need to ensure the integrity, safety, and accurate labeling of the flour using suitable packaging. However, some producers still use raffia bags, thwarting this effort [3,4]. Plastic packaging is a cost-effective solution that fulfills packaging requirements for protection and food distribution, as it prevents physical, chemical, physiological, and microbial deterioration [11,12]. Additionally, plastic enhances the shelf life of packaged foods because it is a moisture and gas barrier, allowing the products to retain their original qualities [13,14].

Polyolefins exhibit low water vapor transmission rates and strong mechanical resistance, which enhance product safety during distribution and preserve the characteristics of the flour during storage [15,16]. Low-density polyethylene and polypropylene are packaging materials widely used for various food products, including other cassava flours; however, to date, no studies have assessed the long-term stability of Copioba cassava flour when packaged in these materials [17,18,19].

LDPE was selected for evaluation as a packaging material based on the custom of local sellers of packing Copioba flour in this material for the regional markets. The PP packaging material was included because of its well-documented good performance as a packaging material for cassava flour. The metallized BOPP packaging material was included because of its potential to preserve the Copioba flour sensorial attributes due to its ability to act as a light barrier, which could improve the preservation of the unique characteristics of Copioba flour, and because it has not yet been assessed for packaging cassava flours.

Notably, no previous study has evaluated the effect of the type of packaging material on the quality of Copioba flour, although there are published studies on other cassava products. None of these cassava products have the same characteristics as Copioba flour [19,20,21,22,23]. Additionally, the general characteristics and traditional production ways of Copioba flour have been documented, but specific characteristics, such as water activity and moisture trajectories, texture/hardness, and color, have not yet been explored [6,7,24,25].

Therefore, this study aimed to evaluate the influence of the type of commercial polyolefin packaging material on some important quality attributes of Copioba cassava flour, such as its texture and color, under controlled environmental conditions. The data collected was envisaged to support its Geographical Indication (GI) and enhance understanding of the practical implications of its packaging.

## 2. Materials and Methods

**Figure 1:**
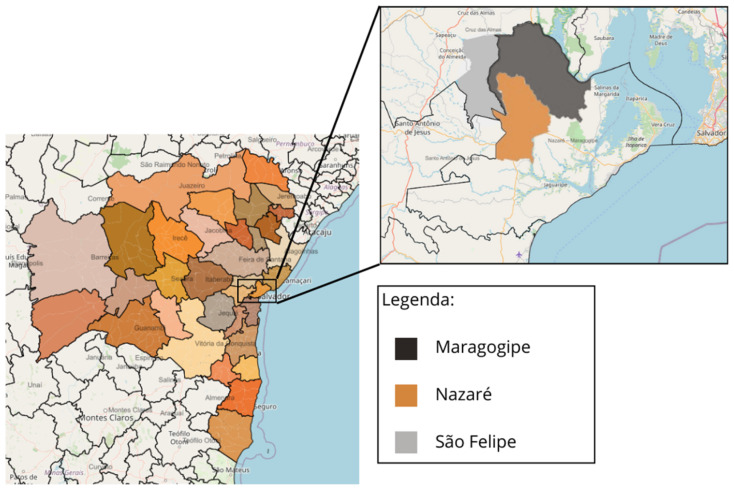
Location of the Copioba Valley region. Source: IBGE [26], adapted.

**Scheme 1:**
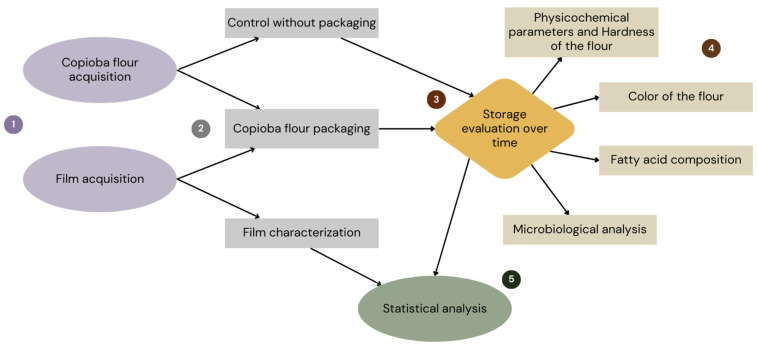
Flowchart of the methodology steps followed in this research.

Polyolefin films, low-density polyethylene (LDPE), polypropylene (PP), and metallized biaxially oriented polypropylene (BOPP) films were obtained from polymeric packaging enterprises Totalflex (Jequié-BA, Brazil), Plaskem (Lauro de Freitas-BA, Brazil), and Embamat (Lauro de Freitas-BA, Brazil). Copioba cassava flour was supplied by a local provider in Nazaré, situated at a latitude of 13°02′06″ S and a longitude of 39°00′52″ W, in Copioba Valley, which encompasses the supposed GI region (Figure 1). Experiments were conducted at the Applied Chromatography Research Laboratory (LAPESCA), located at a latitude of 12°60′00″ S, a longitude of 38°30′29″ W, and an altitude of 14 m above sea level. A flowchart describing the steps followed during this research is provided in Scheme 1.

### 2.1. Characterization of Packaging Material

#### 2.1.1. Thickness

Film thickness was measured using a digital micrometer (Digimess, Derbyshire, UK) with a plain pinch (0–25 mm, resolution of 0.001 mm). The measurements were conducted in a rectangular portion of the films by taking eight random measurements, in triplicate.

#### 2.1.2. Mechanical Properties

The mechanical properties (maximum tension, and maximum and specific deformation) were determined through tests in a Universal Essays Machine (Emic, model DL 200 MF) (São José dos Pinhais, PR, Brazil), with a capacity of 200 Kgf, according to the guidelines in the ASTM D882-18 [27], with a velocity of 12.5 mm/min and at a temperature of 25 °C. For each sample, the traction essays were conducted in 10 test bodies, measuring 100 mm in length and 25 mm in width.

#### 2.1.3. Water Vapor Transmission Rate (WVTR)

Film WVTR was determined according to guidelines in the ASTM E96/E96M-24a [28]. To conduct the measurements, a glass recipe, distilled water, and an impermeable adhesive (fast-dry epoxy resin) were used, and WVTR was calculated using Equation (1) as follows:

(1) $$WVTR\;\left(g/m^{2}\cdot day\right)\;=\;\frac{G}{t\cdot Ap},$$

where *WVTR* = water vapor transmission rate, *G* = mass variation, *t* = time, and *Ap* = permeation area.

### 2.2. Assessment of Storage of Copioba Cassava Flour in Various Packaging Materials

The Copioba cassava flour was characterized before the experiments began. To analyze the effect of packaging on the quality of flour, a 250 g sample of each was placed in various packaging material (LDPE, PP, and BOPP) for sensitivity evaluation, and sealed with a thermostat sealer (Sulpack, Caxias do Sul, Brazil, model SM350), except for the control, which was kept exposed in open Petri plates, as the Copioba flour is traditionally commercialized in that way. The sealed samples were stored at 30 ± 5 °C and a relative humidity of 60 ± 5% in a climatic chamber (Tecnal, Piracicaba, Brazil, model TE-400L); these conditions represent the average climate conditions of several parts of Brazil. The flour samples packaged in various films and the control treatment were observed every 30 days for 120 days, in triplicate for each condition evaluated. The observations were triplicated by preparing three bags containing Copioba cassava flour for each packaging material and time combination.

### 2.3. Measurement of Physicochemical Parameters and Hardness of Copioba Cassava Flour

The granulation of Copioba cassava flour was evaluated by the sieving method, in triplicate [7]. Moisture content (%) was measured in a sample moisture analyzer (AND, Tokyo, Japan, model MX50), in triplicate, adjusting the intensity of the emitted radiation so that the sample reached 105 °C, according to the manufacturer’s instructions. The flour’s water activity (*a*_w_) was determined in triplicate, in an AquaLAB LITE Analyzer (Decagon Devices, Pullman, WA, USA, model AL 1536), which uses the dielectric constant principle for measuring the water activity, according to the manufacturer’s instructions.

About 10 g of each sample was diluted in 25 mL of sterile distilled water before the pH was measured using a pH meter (Hanna Instruments, Woonsocket, RI, USA, model HI 221), in triplicate. Titratable acidity (TA) (meq NaOH/100 g) was determined by titrating 0.1 N sodium hydroxide to 10 g of the sample diluted in 50 mL of distilled water, using phenolphthalein as an indicator, in triplicate [29].

Mechanical strength was measured with a texture analyzer (Stable Micro Systems, Godalming, UK, model TA.XT Express). A flat cylindrical plunger, with a circular probe, 75 mm in diameter, was set to a speed of 1 mm/s, with a distance of 9.5 mm between the probe and the sample. Each sample was measured in 10 replicates. Hardness (gf) was determined during storage and defined as the maximum force at the product’s breaking point [30].

### 2.4. Color of Copioba Cassava Flour

Flour color was evaluated using a colorimeter (Konica Minolta, Tokyo, Japan, model CR-5), against a white background. The measurements were performed in triplicate. For color evaluation, CIELab patterns were used, in which L* ranges from 0 (black) to 100 (white), a* from green (−) to red (+), and b* ranges from blue (−) to yellow (+) [31].

### 2.5. Determination of Fatty Acid Composition and Content

Lipids were extracted by Bligh and Dyer, and fatty acids were separated and identified by gas chromatography [32,33]. An aliquot (25 mg) of the fraction of total lipids was subjected to the transesterification process. For saponification, 1.5 mL of methanolic NaOH (0.5 mol/L) was added to the sample, and the sample was heated at 100 °C for 15 min. After cooling to room temperature, 2 mL of the methanolic solution of BF3 (12%) was added, and the mixture was heated at 100 °C for 30 min. The solution was cooled in a cold water bath. After adding 2 mL of isooctane and stirring for 5 min, 5 mL of saturated sodium chloride solution was added with stirring for 2 min. After phase separation, the upper phase containing the fatty acid methyl esters (FAMEs) was collected and stored under an inert atmosphere (N_2_) at −18 °C until analysis by gas chromatography. Separation of the FAME was performed by gas chromatography in a gas chromatograph (Perkin Elmer Clarus 680, Waltham, MA, USA) equipped with column CP—Wax (25 m × 0.25 mm × 0.20 µm) and a flame ionization detector (GC-DIC). The analysis was performed at an injector temperature of 250 °C and detector temperature of 280 °C. The following thermal profile was used: 150 °C for 16 min, before increasing temperature by 2 °C/min up to 180 °C, and maintained for 25 min; followed by an increase of 5 °C/min up to 210 °C, and maintained for 25 min; and a final increase of 10 °C/min up to 230 °C, which was maintained for 16 min. Helium was used as a carrier gas at 1.0 mL/min. Injections of 1 µL of FAME solutions were performed in triplicate.

The FAMEs were identified by comparing the retention times (Rt) of the peaks of the samples with a mixture of FAME standards (37 FAME C4:0-C24:0, 189-19, Sigma, St. Louis, MO, USA), separated under the same chromatographic conditions, and an internal standard (C23:0 Sigma^®^, USA). The esters were expressed in mg per gram of lipid [34].

The concentration of fatty acids was calculated according to Equation (2) as follows:

(2) $$Concentration\;\left(mg/g\right)\;=\;\frac{A_{x}\cdot W_{is}\cdot {CF}_{x}}{A_{x}\cdot W_{s}\cdot {CF}_{s}}\cdot 1000,$$

where *A_x_* = area of methyl ester fatty acid peek in the chromatogram of the sample, *W_is_* = weight (in mg) of internal standard added to the sample, *CF_x_* = correction factor response of each fatty acid methyl ester ionization detector, relative to C23:0, *A_is_* = area of internal standard methyl ester of fatty acid peak in the chromatogram of the sample, *W_s_* = sample weight (in milligrams), and *CF_s_* = conversion factor of fatty acid methyl ester to fatty acid.

### 2.6. Microbiological Analysis

*Bacillus cereus*, and total yeast and mold counts analysis were performed according to the methodology described by Downes and Ito [35]. After analysis, colonies of the *B. cereus* group were confirmed through anaerobic growth in red glucose broth, tyrosine decomposition, VP (Voges–Proskauer) test, nitrate reduction, and motility [36].

### 2.7. Statistical Analysis

The experiments were conducted in three replicates in a completely randomized design, in a factorial scheme, with time and packaging material being the factors. The data were submitted to analysis of variance, followed by Tukey’s multiple comparison test when necessary. A significance level of 5% was used.

## 3. Results

Sieve analysis showed that the mean Copioba cassava flour particle size was between 0.125 and 0.71 mm, representing a total of 92.27%, with 51.98% being lower than 0.425 mm, indicating that Copioba cassava flour should be classified as a flour of fine granulation (Figure S1).

### 3.1. Packaging Characterization

The results presented in Table 1 indicate the property differences in each film used to pack the flour. The thicknesses of all the LDPE and PP films differed significantly from that of metallized BOPP, which exhibited higher maximum tension, lower deformation, and WVTR values than LDPE and PP (*p* < 0.05).

### 3.2. Effect of Type of Packaging Material on the Physicochemical Parameters and Texture of Copioba Cassava Flour

**Figure 2:**
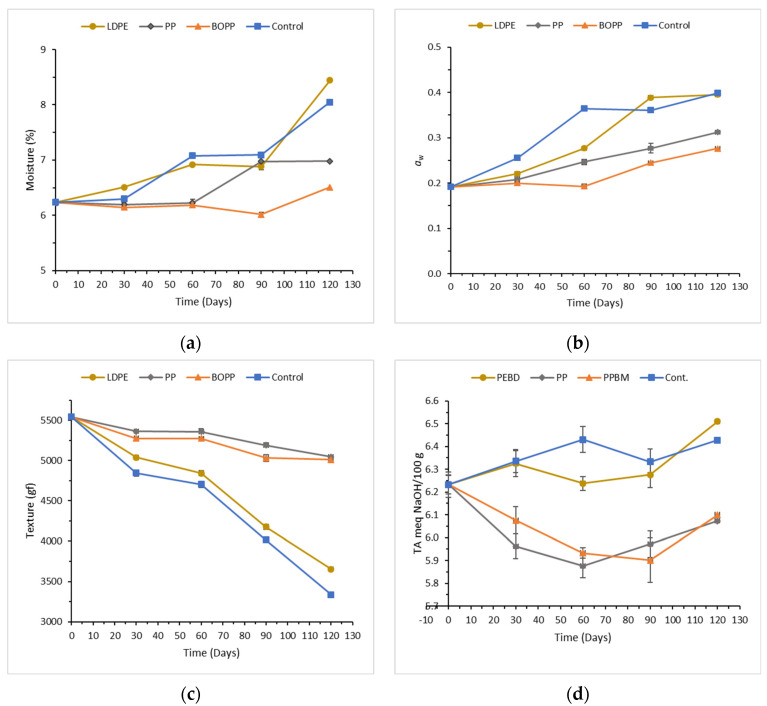
Physicochemical parameters: (**a**) moisture content, (**b**) *a*_w_, (**c**) texture, and (**d**) TA of Copioba cassava flour in various types of packaging material and control (without packaging) during storage.

Moisture content and *a*_w_ increased over time in all types of packaging material and the control (Figure 2). The effect was more pronounced for the control and for the flour packaged in LDPE, and the best result was recorded for the metallized BOPP packaging, in which the changes in *a*_w_ were observed only after 60 days of storage. Additionally, the texture of Copioba flour decreased during storage to different degrees, depending on the type of packaging. The highest reduction (39.76%) occurred in the control (unpackaged), decreasing from 5541.2 ± 30.8 g to 3338.1 ± 13.8 g during storage, indicating the influence of the type of packaging on product stability and shelf life. Furthermore, the titratable acidity (TA) changed only after 90 days of storage for the flour packaged in LDPE, and was almost constant for the flour packaged in PP and BOPP after a decrease in the first 30 days of storage, with a slight variation observed for those packaged in BOPP between 90 and 120 days of storage. The TA of the flour stored without packaging (control) did not vary over time, remaining highest among the types of packaging evaluated (*p* < 0.05) (Figure 2 and Table S1).

Notably, the pH of the Copioba flour in all types of packaging and the control showed similar fluctuations during storage, with slight but statistically significant differences (*p* < 0.05) at the end of storage (120 days). It varied from 4.84 ± 0.02 to 5.16 ± 0.03, a range which is too small to be practically important and is in the range reported in previous studies (Table S1 and Figure S2) [19,37]. The TA values of the flour packaged in PP and metallized BOPP were not significantly different (*p* < 0.05) at all evaluated times, ranging from 5.8756 ± 0.0542 to 6.1001 ± 0.0971 meq NaOH/100 g. A similar trend was observed between the flour packaged in LDPE and the control, with no significant difference (*p* < 0.05) at almost all evaluated times, and values varied from 6.2383 ± 0.0579 to 6.5098 ± 0.0559 meq NaOH/100 g (Table S1).

Changes in moisture content, *a*_w_, and texture were higher in the flour without packaging (control) and that packaged in LDPE, than in the flour packaged in PP and metallized BOPP films. This highlights the importance of the type of packaging material used in stabilizing the product during storage, which is important in adding value to the production chain and conquering new markets.

### 3.3. Effect of Storage Time on the Color of Copioba Cassava Flour

**Figure 3:**
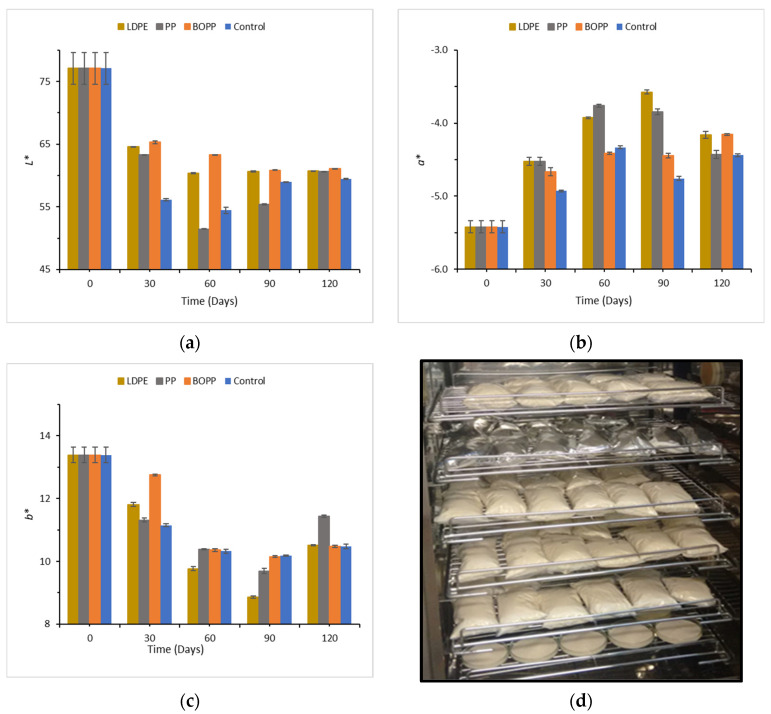
Colorimetric parameters of Copioba cassava flour packaged in various types of packaging material and control (without packaging) during storage: (**a**) L* coordinate, (**b**) a* coordinate, (**c**) b* coordinate, and (**d**) Copioba flour placed in the dryer.

The influence of storage time on color parameters is shown in Figure 3. The results showed that all parameters varied during storage time, with a decrease in the L* parameter, indicating that the flour turned darker over time. A similar behavior was observed for the b* parameter.

The parameter a* did not differ significantly (*p* < 0.05) between 30 and 120 days of storage (Table S2). At the end of storage (120 days), the flour packaged in PP presented the highest value for the parameter b*.

### 3.4. Effect on Fatty Acid Composition

Despite the low content of total lipids in Copioba cassava flour (≤1.0%), the baseline contents of unsaturated fatty acids (≈70%) and polyunsaturated fatty acids (≈34% C18:2n6, C18:3n3 at baseline) were high (Table S3). These lipids have a higher possibility of oxidation and, consequently, a high ability to reduce the sensorial quality and nutritional factor of Copioba cassava flour, regardless of the storage conditions [7].

**Figure 4:**
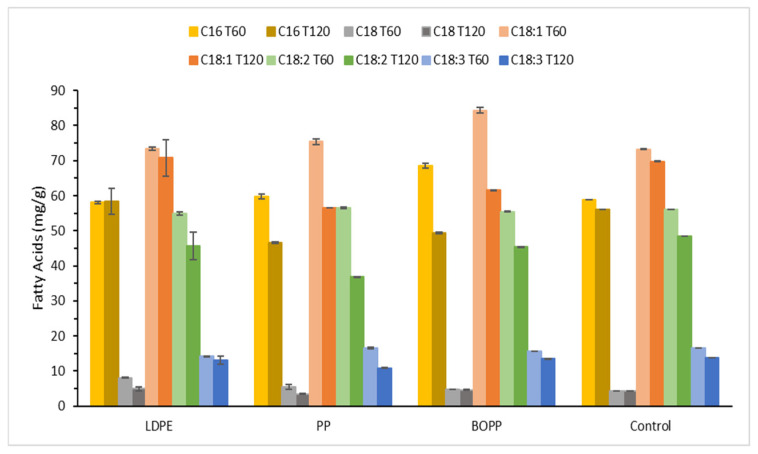
Effect of type of packaging material on the fatty acid content [mg/g] of Copioba cassava flour.

The reduction in fatty acid content ranged from 55.81 to 68.28% in the flour after 120 days of storage, depending on the type of packaging material (packaging or control); the decrease was less pronounced between 60 and 120 days of storage (Figure 4 and Table S3).

### 3.5. Microbiological Analysis

The results of yeast and mold and *B. cereus* growth in flour stored in various containers and unpackaged (control) for up to 120 days are shown in Table 2.

A typical behavior of microbial growth was observed for the total yeast and molds during the 120 days of flour storage (Table 2).

After 120 days of storage, the yeast and mold cell count increased by about 50% for the flour packaged in PP, 65% for the flour packaged in metallized BOPP, 100% for the flour packaged in LDPE, and 138% for the control treatment. For *B. cereus*, which was the safety target microorganism chosen as it is a cause for especially for this type of product, counts in flour ranged from <1 log CFU/g to 3.47 log CFU/g (Table 2). Its count in the flour increased 100% from day zero to 120 days of storage in PP and metallized BOPP packaging, whereas in LDPE packaging and the control, the count increased by about 300%, reaching a level above that of 3 log CFU/g, the maximum allowed for safe commercialization in Brazil [38].

Among the packaged and unpackaged flour, PP and metallized BOPP films showed the best results, 3 log CFU/g and 3.3 log CFU/g of yeast and mold count, respectively, after 120 days of storage. Thus, PP and metallized BOPP packaging were more effective in protecting the product than the control, which showed a strong growth of yeast and mold.

## 4. Discussion

The particle size of Copioba flour recorded in this study (Figure S1) falls within the size range classified as thin cassava (cassava flour with particles retained in sieves with openings below 2 mm) in the national legislation [39]. The particle size range reported in previous studies for Copioba flour was retained mostly in sieves with openings of 0.25 mm and below. However, the average retention percentage obtained is in accordance with the results for Copioba flours acquired outside the Copioba Valley [7,24]. The particle size distribution is an important parameter as it determines flour texture and water mass transport during drying. Smaller particle sizes lead to flour with higher crunchiness and a more attractive color [7].

During flour transportation and commercialization, the mechanical properties of the packaging films, such as tension and deformation, are important because they determine the ability of the packaging film to resist damage from impacts and friction, which may influence the product’s sensorial characteristics. Additionally, damage to packaging may increase the gases and product interactions, which diminish flour quality. BOPP presented the lowest maximum and specific deformation values, indicating that it has a competitive advantage over the other films evaluated for packaging Copioba cassava flour.

In this study, the Copioba cassava flour had a mean moisture content of 6.24 ± 0.05%, peaking at 8.44 ± 0.06% at the end of the storage time in flour packaged in LDPE (Table S1). The maximum moisture content of 8.44 ± 0.06% recorded in this study is below the legislated maximum limit (<15%) for general flour [40]. The moisture content values recorded in this study were in the range of those reported by Matos et al. [24] for denominated Copioba cassava flours, which varied from 4.65 to 7.98%. However, Pascoal et al. [7] recorded low moisture content varying from 0.96 to 8.42% Copioba cassava flour, most probably due to high roasting temperatures. Aryee et al. [37] reported higher moisture content values for unpackaged flour in 31 cassava cultivars, varying from 3.21 to 11.75%. In this case, the flour was dried at 50–55 °C for over days. These findings indicate that the moisture content of cassava flour is influenced by many factors, such as source, location, season, and processing.

Chukwu and Abdullahi [41] reported an increase in moisture content from 3.50 to 5.69% in cassava flour from Nigeria, which had been dried at 85 °C over 24 h and packaged in polyethylene film for three weeks. This was a more pronounced variation in moisture content than that recorded in this study (6.23 to 6.40%, Figure 2a). According to the authors, the increase in moisture content could have been a result of variation in storage temperature and relative humidity, which were not controlled, unlike in this study, in which the temperature and humidity were maintained at 30 ± 5 °C and 60 ± 5%, respectively.

Opara et al. [19] found a negative variation in moisture content in cassava flour in 12 weeks of storage in all the types of packaging evaluated, including LDPE, plastic buckets, and paper bags, with values varying from 12 to 10.9%, in a controlled chamber at 23 °C and 60% humidity.

In this study, *a*_w_ and moisture content showed significant variations among the types of packaging material, with an increase in moisture content and water activity (*a*_w_) in the cassava flour being observed during storage (Figure 2). Owing to the hygroscopic nature of the flour, the change in *a*_w_ relates to the variation in the level of humidity during the storage period [17].

The WVTR of packaging materials is an important criterion for predicting moisture absorption by food. It influences a_w_ of the Copioba flour as it transports water particles by capillarity [42]. The results demonstrated the best control of moisture content and *a*_w_ in the Copioba flour packaged in metallized BOPP, followed by that packaged in PP. Polyolefins are excellent barriers to moisture, and BOPP performs better than the non-oriented PP, because the orientation of molecules reduces the intermolecular space available for diffusion [43]. Lazić et al. [44] reported that metallization of BOPP with aluminum can provide up to a twofold improvement in barrier characteristics against water vapor. These findings support the observed behavior of moisture and water activity (*a*_w_) in Copioba flour documented in this study.

The texture of Copioba cassava flour, known for its crispness, is attributed to its low moisture content, achieved through a gradual increase in roasting temperature, and to the flour’s small particle size, which increases surface area, thereby improving the water mass transfer in its evaporation. This characteristic must be retained during storage time because it is one of the most important sensorial parameters for the Copioba cassava flour [3,7,24]. Therefore, the observed change in texture, indicated by the reduction in the hardness of cassava flour, was probably caused by changes in *a*_w_ and moisture content. It was observed that cassava flour packaged in materials with higher WVTR showed a greater reduction in hardness. This was expected because low hardness leads to an increase in *a*_w_ and moisture content sufficient to fill the free volume (at a microscopic level), leading to a higher interaction between water and other molecular components. A similar behavior of cassava flour was reported by Kulchan et al. [45].

Additionally, storage conditions, roasting temperature, composition of Copioba cassava flour, and *a*_w_ may have contributed to the characteristic color of the Copioba flour [46,47]. Variation in the color parameters observed during storage presented a similar behavior in the flour regardless of the type of packaging material.

According to the local legislation, the TA value for the Copioba flour recorded in this study is on a scale of flours with high acidity (TA over 3 meq NaOH/100 g for a dry cassava flour) [39]. In the production of Copioba cassava flour, comminuted pasta is exposed for an extended period at room temperature, mainly on the pressing step, resulting in spontaneous fermentation and, consequently, in high acidity of the product, which is one of the sensorial attributes for this type of flour [4,6,7]. In this study, the initial TA value was 6.23 ± 0.06 meq NaOH/100 g, which is within the range observed in previous studies, in which values ranging from 1.81 to 6.53 meq NaOH/100 g were reported [7,40]. After 120 days of storage, TA values ranged from 6.07 ± 0.06 to 6.51 ± 0.06 meq NaOH/100 g, with an average reduction in TA of 2.33% for the flours packaged in PP and metallized BOPP and an average increase of 3.85% for the flours packaged in LDPE and in the control. These high values for TA recorded in this study indicate the importance of evaluating other forms of packaging the Copioba cassava flour to preserve its integrity and quality.

Another parameter evaluated as a quality standard for Copioba flour is its color, which is used by consumers to identify flours from the Copioba Valley. Regardless of the variations observed in the associated component a*, which depends on the cassava variation used to produce the Copioba flour, the values for parameters b* and L* indicated a tendency to a white-yellowish tone, its characteristic color, preserved with slight variations at the end of 120 days of storage (Figure 3). Oliveira et al. [6] recorded a similar yellow tone in their study of 26 samples of cassava flour produced artisanally by the rural community of Baixada Cuiabana region, in the west-central region of Brazil. The intensity of Copioba flour color is related to processing, which may favor non-enzymatic browning by the Maillard or browning reaction, as the Copioba flour has more than 80% starch content [25].

The reduction in fatty acid content might have caused a decrease in Copioba flour sensory quality, as lipid oxidation may be correlated with the formation of off-flavors as the amount of volatile compounds increased, as observed by Pascoal et al. [7]. There were no significant differences between the types of packaging material in preserving fatty acid content over time [48].

Opara et al. [19] recorded a reduction in fat content during storage in cassava flours of two cultivars and attributed it to lipid enzymes. They observed a 20 to 33% reduction in fat content depending on the variety and type of packaging. As the lipid content in the flour was low, even a small amount of oxygen available would be enough for its oxidation. Notwithstanding, according to Stewart-Jones et al. [49], the change in fatty acid content may suggest that the cassava flour contains enzymes (lipases) that hydrolyze triglycerides.

The yeast and mold count in Copioba cassava flour was higher than the bacterial count at the end of the storage period, which may be a consequence of the ability of the yeasts and molds to withstand adverse environmental conditions. Similar results have been reported elsewhere [20,41].

The reproduction and growth rate of yeast, molds, and bacteria affect the quality of store cassava flour. The reproduction and growth of microbes (yeast, molds, and bacteria) depend on environmental variables, mainly moisture content and *a*_w_. Different packaging material protects cassava flour in various ways depending on the properties of the packaging material. Therefore, the constant increase in the counts of yeast and mold, and *Bacillus cereus* in Copioba cassava flour stored in various types of packaging was consistent with the moisture content and *a*_w_ achieved in each packaging at the end of storage.

## 5. Conclusions

To the best of our knowledge, this study was the first attempt to evaluate the influence of the type of packaging material on the stability and maintenance of the identity of Copioba cassava flour. Additionally, the results are important because they contribute to the Geographic Indication of the product. The increases in water activity and moisture content were the critical parameters that influenced the change in quality attributes of Copioba cassava flour after 120 days of storage. Thus, PP or metallized BOPP are recommended for packaging Copioba cassava flour, because they can protect the product, the producer, and the consumer, thereby adding value to the entire production chain. Additionally, these packaging materials allow the use of nutritional information and graphical representation of the GI on the label.

## Figures and Tables

**Table 1:** Characterization of thickness, mechanical properties, and water vapor transmission rate of commercial materials used to package Copioba cassava flour in this study.

Packaging Material	Thickness (mm)	Maximum Tension (MPa)	Maximum Deformation (mm)	Specific Deformation (mm/mm)	WVTR (g/m^2^∙day)
LDPE	0.099 ± 0.009 ^a^	28.00 ± 3.61 ^a^	426.33 ± 61.53 ^a^	8.33 ± 1.53 ^a^	12.10 ± 2.24 ^a^
PP	0.084 ± 0.009 ^a^	38.33 ± 1.15 ^b^	266.33 ± 3.79 ^b^	5.00 ± 0.00 ^b^	10.79 ± 2.57 ^a^
BOPP	0.050 ± 0.006 ^b^	35.00 ± 0.00 ^b^	33.67 ± 2.52 ^c^	1.00 ± 0.00 ^c^	2.93 ± 0.34 ^b^

The means with the same letters in the same columns presented no significant difference (*p* > 0.05) according to Tukey’s test.

**Table 2:** Monitoring microbial growth in Copioba cassava flour packaged in various types of packaging during storage [log CFU/g].

Microorganism	Packaging Material	T = 0	T = 30 Days	T = 60 Days	T = 90 Days	T = 120 Days
Yeast and molds	LDPE	2	2.30	3.47	4.69	4
	PP		2	2.60	3	3
	BOPP		2	2	3.30	3.30
	CONTROL		2.90	3	4	4.77
*B. cereus*	LDPE	<1	2.30	3.30	2.60	3.30
	PP		2	2.30	2	2
	BOPP		< 1	< 1	1.60	2
	CONTROL		2.30	3	2.77	3.47

## Data Availability

The original contributions presented in this study are included in the article/Supplementary Material. Further inquiries can be directed to the corresponding authors.

## Supplementary Materials

The following supporting information can be downloaded at: https://www.mdpi.com/article/10.3390/ma18204768/s1, Table S1. Mean Test between physicochemical parameters of Copioba cassava flour over storage time. Table S2. Mean Test between colorimetric parameters of Copioba cassava flour over storage time. Table S3. Effect of different packaging on the range of fatty acids [mg/g] of Copioba cassava flour. Figure S1. pH values of Copioba cassava flour in different packaging and control (without packaging). Figure S2. pH values of Copioba cassava flour in different packaging and control (without packaging).

## Abbreviations

The following abbreviations are used in this manuscript:

LDPE	Low-density polyethylene
PP	Polypropylene
BOPP	Metallized biaxially oriented polypropylene
*a*_w_	Water activity
TA	Titratable acidity
CFU	Colony forming unit

## References

[1] FAOSTAT (2023). Food and Agriculture Organization Corporate Statistical Database.

[2] IBGE (2023). Instituto Brasileiro de Geografia e Estatística—Produção Agropecuária.

[3] Silva S.B., Pena L.C.C., Cardoso R.C.V. (2023). Copioba ad common cassava flour know-how: Establishing similarities and distinctions in São Felipe, Brazil. Int. J. Gastron. Food Sci..

[4] Silva I.R.C., Cardoso R.C.V., Góes J.A.W., Druzian J.I., Vidal Júnior P.O., Andrade A.C.B. (2017). Food safety in cassava “flour houses” of Copioba valley, Bahia, Brazil: Diagnosis and contribution to geographical indication. Food Control.

[5] de Oliveira O.S., Brito V.H.S., Cereda M.P. (2018). Establishing a standard for handmade Brazilian cassava flour from Baixada Cuiabana (Mato Grosso, Brazil) to support its processing and sale. Food Sci. Technol..

[6] Freitas-Sá D.G.C., Teixeira K.T.R., Mattos C.T.G.B., Monteiro R.P. Atributos de aparência da farinha de copioba da Bahia como contribuição à indicação geográfica. Proceedings of the XXV Congresso Brasileiro de Ciência e Tecnologia de Alimentos.

[7] Pascoal D.R.C., Moura L.E., da Silva J.R., Assis D.J., Costa S.S., Druzian J.I. (2022). Characteristics volatiles of cassava flours and their relationship to parameters other, process and geographical origin: A preliminary study. Food Sci. Technol..

[8] Su S., Wang L., Feng C., Liu Y., Li C., Du H., Tang Z., Xu Y., Wang L. (2016). Fingerprints of anthocyanins and flavonols of *Vaccinium uliginosum* berries from different geographical origins in the Greater Khingan Mountains and their antioxidant capacities. Food Control.

[9] Crescenzi R., Filippis F., Giua M., Vaquero-Piñero C. (2022). Geographical indications and local development: The strength of territorial embeddedness. Reg. Stud..

[10] INPI (2025). Instituto Nacional de Propriedade Industrial. Indicações Geográficas: Indicações de Procedências Reconhecidas.

[11] Chea V., Angellier-Coussy H., Peyron S., Kemmer D., Gontard N. (2016). Poly(3-hydroxybutyrate-co-3-hydroxyvalerate) films for food packaging: Physical–chemical and structural stability under food contact conditions. J. Appl. Polym. Sci..

[12] Siddiqui S.A., Yang X., Deshmukh R.K., Gaikwad K.K., Bahmid N.A., Castro-Muñoz R. (2024). Recent advances in reinforced bioplastics for food packaging—A critical review. Int. J. Biol. Macromol..

[13] Dey A., Dhumal C.V., Sengupta P., Kumar A., Pramanik N.K., Amal T. (2021). Challenges and possible solutions to mitigate the problems of single-use plastics used for packaging food items: A review. J. Food Sci. Technol..

[14] Awol S.M., Kuyu C.G., Bereka T.Y. (2023). Physicochemical stability, microbial growth, and sensory quality of teff flour as affected by packaging materials during storage. LWT.

[15] Macedo I.S.M., Sousa-Gallagher M.J., Oliveira J.C., Byrne E.P. (2013). Quality by design for packaging of granola breakfast product. Food Control.

[16] Opara U.L., Mditshwa A. (2013). A review on the role of packaging in securing food system: Adding value to food products and reducing losses and waste. Afr. J. Agric. Res..

[17] Agrahar-Murugkar D., Jha K. (2011). Influence of storage and packaging conditions on the quality of soy flour from sprouted soybean. J. Food Sci. Technol..

[18] Golestan M.N., Ghosta Y., Pourmirza A.A., Valizadegan O. (2015). Study on laser perforated films as gas permeable packaging for confused flour beetle control inside food packaging. J. Stored Prod. Res..

[19] Opara U.L., Caleb O.J., Uchechukwu-Agua A.D. (2016). Evaluating the Impacts of Selected Packaging Materials on the Quality Attributes of Cassava Flour (cvs. TME 419 and UMUCASS 36). J. Food Sci..

[20] Adejumo B.A., Raji A.O. (2012). Microbiological safety and sensory attributes of garri in selected packaging materials. Acad. Res. Int..

[21] Ogiehor I.S., Ikenebomeh M.J. (2006). The effects of different packaging materials on the shelf stability of garri. Afr. J. Biotechnol..

[22] Uchechukwu-Agua A.D., Caleb O.J., Manley M., Opara U.L. (2015). Effects of storage conditions and duration on physicochemical and microbial quality of the flour of two cassava cultivars (TME 419 and UMUCASS 36). CyTA J. Food.

[23] Ekeledo E., Abass A., Müller J. (2024). Effect of packaging and storage conditions on the pasting and functional properties of pretreated yellow-fleshed cassava flour. Appl. Food Res..

[24] de Matos M.F.R., da Silva I.R.C., Mendonça T.A., Santos L.F.P., Nunes I.L., Druzian J.I. (2012). Conformity of cassava flours type copioba commercialized in the fairs in salvador (BA) with the parameters of the legislation: A contribution to geographical indication (GI) of the product. Rev. Geintec.

[25] Silva A.C.M.S., Pinho L.S., Sousa L.S., Moura L.E., Souza C.O., Druzian J.I. (2015). Classificação, identidade e matérias estranhas de farinha de mandioca copioba: Conformidade com a legislação brasileira e contribuição a indicação geográfica. Cad. Prospec..

[26] IBGE (2025). Instituto Brasileiro de Geografia e Estatística—Portal de Mapas.

[27] (2018). Standard Test Method for Tensile Properties of Thin Plastic Sheeting.

[28] (2014). Standard Test Methods for Gravimetric Determination of Water Vapor Transmission Rate of Materials.

[29] AOAC (2000). Official Methods of Analysis.

[30] Li Y., Kloeppel K.M., Hsieh F. (1998). Texture of glassy corn cakes as a function of moisture content. J. Food Sci..

[31] Bible B.B., Singha S. (1993). Canopy position influences CIELAB coordinates of peach color. HortScience.

[32] Bligh E.G., Dyer W.J. (1959). A Rapid method of total lipid extraction and purification. Can. J. Biochem. Physiol..

[33] Joseph J.D., Ackman R.G. (1992). Capillary column gas chromatography method for analysis of encapsulated fish oil and fish oil ethyl esters: Collaborative study. J. AOAC Int..

[34] Nascimento I.A., Marques S.S.I., Cabanelas I.T.D., Pereira S.A., Druzian J.I., Souza C.O., Vich D.V., Carvalho G.C., Nascimento M.A. (2013). Screening microalgae strains for biodiesel production: Lipid productivity and estimation of fuel quality based on fatty acids profiles. Bioenergy Res..

[35] Downes F.P., Ito K. (2001). Compendium of Methods for the Microbiological Examination of Foods.

[36] FDA (1998). Bacteriological Analytical Manual.

[37] Aryee F.N.A., Oduro I., Ellis W.O., Afuakwa J.J. (2006). The physicochemical properties of flour samples from the roots of 31 varieties of cassava. Food Control.

[38] Agência Nacional de Vigilância Sanitária (ANVISA) (2001). Resolução de Diretoria Colegiada Nº 12.

[39] MAPA (2011). Ministério da Agricultura, Pecuária e Abastecimento, Instrução Normativa 52/2011.

[40] ANVISA (2022). Agência Nacional de Vigilância Sanitária, Resolução da Diretoria Colegiada—RDC 711/2022.

[41] Chukwu O., Abdullahi H. (2015). Effects of moisture content and storage period on proximate composition, microbial counts and total carotenoids of cassava flour. Int. J. Innov. Sci. Eng. Technol..

[42] Famurewa J.A.V., Oluwamukomi M.O., Alaba J.O. (2012). Storage stability of pupuru flour (a cassava product) at room temperature. Br. J. Appl. Sci. Technol..

[43] Massey L.K. (2003). Permeability Properties of Plastics and Elastomers.

[44] Lazić V., Budinski-Simendić J., Gvozdenović J., Simendić B. (2010). Barrier properties of coated and laminated polyolefin films for food packaging. Acta Phys. Pol. A.

[45] Kulchan R., Boonsupthip W., Suppakul P. (2010). Shelf life prediction of packaged cassava-flour-based baked product using empirical models and activation energy for water vapor permeability of polyolefin films. J. Food Eng..

[46] Chinnici F., Guerrero E.D., Sonni F., Natali N., Marín R.N., Riponi C. (2009). GC-MS characterization of volatile compounds in quality vinegars with protected European geographical indication. J. Agric. Food Chem..

[47] Nomi Y., Masuzaki R., Terasawa N., Takenaka M., Ono H., Otsuka Y., Murata M. (2013). Formation mechanism and characterization of dilysyldipyrrolones, the Maillard-type yellow pigments. Food Funct..

[48] Robertson G.L. (2005). Food Packaging: Principles and Practice.

[49] Stewart-Jones A., Stirrup T.J., Hodges R.J., Farman D.I., Hall D.R. (2009). Analysis of free fatty acids in food substrates and in the dust and frass of stored-product pests: Potential for species discrimination?. J. Stored Prod. Res..

