# Supplementary material for: The Effect of Various Types of Polymeric Packaging Materials on the Quality of Copioba Cassava Flour
Source: Materials (Basel). 2025 Oct 17;18(20):4768. doi: 10.3390/ma18204768 (PMC12566478; doi:10.3390/ma18204768)
Supplement: Supplementary file 1 [file materials-18-04768-s001.zip › materials-3865964-supplementary.pdf]

Table S1. Mean Test between physicochemical parameters of Copioba cassava flour over storage time.

Parameter	Packaging material	T = 0	T = 30 days	T = 60 days	T = 90 days	T = 120 days
Moisture (%)	LDPE	6.24 ± 0.05	6.51± 0.06[ab]	6.92± 0.02[a]	6.88± 0.03[a]	8.44 ± 0.06[a]
	PP		6.19± 0.02[a]	6.23± 0.04[b]	6.97± 0.06[ac]	6.98± 0.07[b]
	BOPP		6.14± 0.05[a]	6.19± 0.02[b]	6.01± 0.02[b]	6.51± 0.05[c]
	CONTROL		6.30± 0.04[b]	7.08± 0.02[c]	7.09± 0.06[c]	8.05± 0.05[d]
a_w	LDPE	0.192 ± 0.001	0.220± 0.002[a]	0.277± 0.005[a]	0.389± 0.004[a]	0.395± 0.002[a]
	PP		0.208± 0.006[b]	0.247± 0.001[b]	0.277± 0.006[b]	0.312± 0.011[b]
	BOPP		0.200± 0.004[b]	0.193± 0.003[c]	0.245± 0.005[c]	0.276± 0.002[c]
	CONTROL		0.256± 0.003[c]	0.365± 0.003[d]	0.361± 0.004[d]	0.399± 0.001[a]
Texture (gf)	LDPE	5541.2 ± 30.8	5037.9± 31.1[a]	4841.6± 2.7[a]	4178.1± 30.9[a]	3653.9± 30.7[a]
	PP		5363.7± 34.0[b]	5358.5± 18.8[b]	5192.0± 40.3[b]	5045.1± 26.5[b]
	BOPP		5275.3± 9.9[c]	5274.8± 14.0[c]	5032.3± 15.3[c]	5010.5± 42.4[b]
	CONTROL		4844.7± 35.5[d]	4704.1± 37.3[d]	4015.2± 36.9[d]	3338.1± 13.8[c]
pH	LDPE	4.90 ± 0.02	4.95± 0.05[a]	4.99± 0.02[a]	4.85± 0.01[ab]	4.93 ± 0.01[a]
	PP		5.07± 0.06[ab]	5.01± 0.01[a]	4.84± 0.02[ab]	4.90± 0.01[b]
	BOPP		5.03± 0.05[ab]	5.00± 0.01[a]	4.93± 0.06[b]	5.01± 0.01[c]
	CONTROL		5.15± 0.05[b]	5.16± 0.03[b]	4.83± 0.03[a]	4.92± 0.01[a]
TA (meq NaOH/100 g)	LDPE	6.2330 ± 0.0559	6.3247± 0.0426[a]	6.2383± 0.0579[a]	6.2760± 0.0305[a]	6.5098± 0.0559[a]
	PP		5.9622± 0.0404[b]	5.8756± 0.0542[b]	5.9718± 0.0502[b]	6.0733± 0.0576[b]
	BOPP		6.0764± 0.0559[b]	5.9331± 0.0592[b]	5.9016± 0.0223[b]	6.1001± 0.0971[b]
	CONTROL		6.3358± 0.0561[a]	6.4305± 0.0502[c]	6.3329± 0.0566[a]	6.4276± 0.0552[a]

Values in the same column for the same parameter with the same letters present no significant differences ($p < 0.05$).

Table S2. Mean Test between colorimetric parameters of Copioba cassava flour over storage time.

Parameter	Packaging material	T = 0	T = 30 days	T = 60 days	T = 90 days	T = 120 days
*L**	LDPE	77.08 ± 2.56	64.55± 0.02[a]	60.33± 0.08[a]	60.65± 0.08[a]	60.70 ± 0.05[a]
	PP		63.28± 0.07[b]	51.47± 0.07[b]	55.37± 0.08[b]	60.62± 0.06[a]
	BOPP		65.32± 0.23[b]	63.30± 0.04[c]	60.85± 0.05[a]	61.06± 0.04[b]
	CONTROL		56.14± 0.15[c]	54.45± 0.50[d]	58.94± 0.09[c]	59.44± 0.09[c]
*a**	LDPE	-5.42 ± 0.08	-4.53± 0.06[a]	-3.93± 0.01[a]	-3.57± 0.03[a]	-4.16± 0.05[a]
	PP		-4.53± 0.06[a]	-3.76± 0.02[b]	-3.84± 0.04[b]	-4.43± 0.06[b]
	BOPP		-4.67± 0.06[b]	-4.41± 0.02[c]	-4.44± 0.03[c]	-4.15± 0.01[a]
	CONTROL		-4.93± 0.01[c]	-4.33± 0.02[d]	-4.76± 0.03[d]	-4.44± 0.02[b]
*b**	LDPE	13.39 ± 0.24	11.81± 0.06[a]	9.77± 0.06[a]	8.86± 0.04[a]	10.52± 0.02[a]
	PP		11.31± 0.06[b]	10.38± 0.02[b]	9.69± 0.08[b]	11.44± 0.04[b]
	BOPP		12.75± 0.03[c]	10.36± 0.04[b]	10.16± 0.03[c]	10.47± 0.04[a]
	CONTROL		11.15± 0.05[d]	10.32± 0.06[b]	10.18± 0.02[c]	10.48± 0.08[a]

Values in the same column for the same parameter with the same letters present no significant differences ($p < 0.05$).

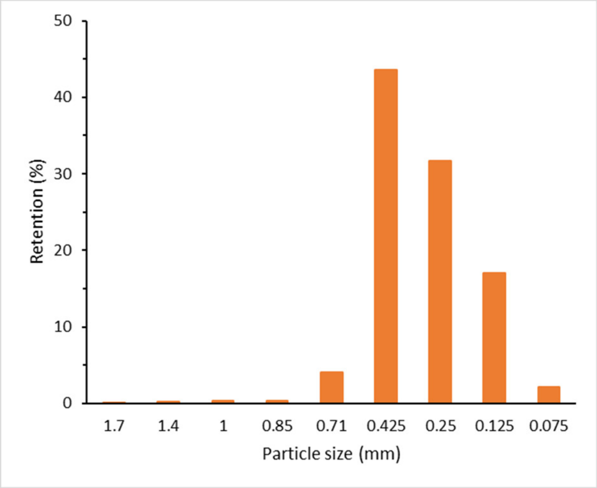

Figure S1. pH values of Copioba cassava flour in different packaging and control (without packaging).

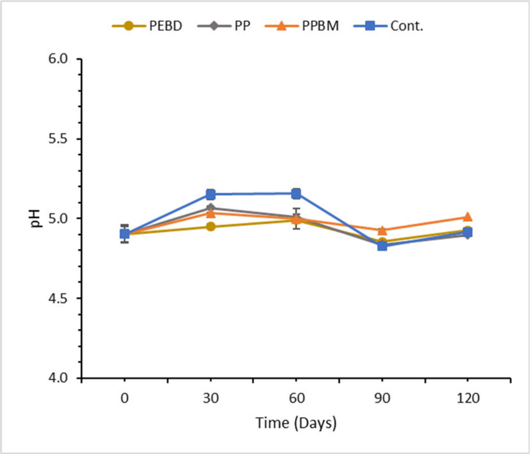

Figure S2. pH values of Copioba cassava flour in different packaging and control (without packaging).

Table S3. Effect of different packaging on the range of fatty acids [mg/g] of Copioba cassava flour.

Fatty acid	Packaging material	T = 0	T = 60 days	T = 120 days
C16:0 Palmitic acid	LDPE	129.43 ± 2.10	58.04 ± 0.36[a]	58.44 ± 3.75[a]
	PP		59.73 ± 0.69[b]	46.55 ± 0.22[b]
	BOPP		68.54 ± 0.65[c]	49.41 ± 0.15[b]
	CONTROL		58.88 ± 0.06[ab]	56.06 ± 0.01[a]
C18:0 Stearic acid	LDPE	13.98 ± 3.80	8.15 ± 0.03[a]	4.94 ± 0.60[a]
	PP		5.40 ± 0.71[b]	3.51 ± 0.04[b]
	BOPP		4.77 ± 0.06[bc]	4.68 ± 0.02[a]
	CONTROL		4.33 ± 0.05[c]	4.36 ± 0.02[a]
C18:1 Oleic acid	LDPE	164.85 ± 1.78	73.50 ± 0.45[a]	70.76 ± 5.26[a]
	PP		75.38 ± 0.84[b]	56.48 ± 0.04[b]
	BOPP		84.31 ± 0.80[c]	61.52 ± 0.09[b]
	CONTROL		73.26 ± 0.16[a]	69.80 ± 0.04[a]
C18:2 Linoleic acid	LDPE	124.22 ± 1.17	54.91 ± 0.36[a]	45.70 ± 4.01[a]
	PP		56.57 ± 0.23[b]	36.79 ± 0.09[b]
	BOPP		55.46 ± 0.17[a]	45.34 ± 0.09[a]
	CONTROL		56.12 ± 0.07[b]	48.44 ±0.05[a]
C18:3 α-linolenic acid	LDPE	35.18 ± 0.38	14.17 ± 0.12[a]	13.22 ± 1.16[a]
	PP		16.58 ± 0.22[b]	10.86 ± 0.15[b]
	BOPP		15.68 ± 0.05[c]	13.50 ± 0.14[a]
	CONTROL		16.52 ± 0.01[b]	13.75 ± 0.03[a]

Values in the same column for the same fatty acid with the same letters present no significant differences ($p < 0.05$).
